# The COPD-SIB: a newly developed disease-specific item bank to measure health-related quality of life in patients with chronic obstructive pulmonary disease

**DOI:** 10.1186/s12955-016-0500-0

**Published:** 2016-06-27

**Authors:** Muirne C. S. Paap, Lonneke I. M. Lenferink, Nadine Herzog, Karel A. Kroeze, Job van der Palen

**Affiliations:** Centre for Educational Measurement at the University of Oslo (CEMO), Faculty of Educational Sciences, University of Oslo, Oslo, Norway; Department of Research Methodology, Measurement, and Data-Analysis, Behavioural, Management and Social Sciences, University of Twente, P.O. Box 217, 7500 AE Enschede, The Netherlands; Department of Clinical Psychology, Behavioural and Social Sciences, University of Groningen, Groningen, The Netherlands; University of Twente, Enschede, The Netherlands; Medical School Twente, Medisch Spectrum Twente, Enschede, The Netherlands

**Keywords:** Item response theory, IRT, Patient perspective, Item bank, COPD, SGRQ-C, MRF-26, VQ11, QoL-RIQ

## Abstract

**Background:**

Health-related quality of life (HRQoL) is widely used as an outcome measure in the evaluation of treatment interventions in patients with chronic obstructive pulmonary disease (COPD). In order to address challenges associated with existing fixed-length measures (e.g., too long to be used routinely, too short to ensure both content validity and reliability), a COPD-specific item bank (COPD-SIB) was developed.

**Methods:**

Items were selected based on literature review and interviews with Dutch COPD patients, with a strong focus on both content validity and item comprehension. The psychometric quality of the item bank was evaluated using Mokken Scale Analysis and parametric Item Response Theory, using data of 666 COPD patients.

**Results:**

The final item bank contains 46 items that form a strong scale, tapping into eight important themes that were identified based on literature review and patient interviews: Coping with disease/symptoms, adaptability; Autonomy; Anxiety about the course/end-state of the disease, hopelessness; Positive psychological functioning; Situations triggering or enhancing breathing problems; Symptoms; Activity; Impact.

**Conclusions:**

The 46-item COPD-SIB has good psychometric properties and content validity. Items are available in Dutch and English. The COPD-SIB can be used as a stand-alone instrument, or to inform computerised adaptive testing.

**Electronic supplementary material:**

The online version of this article (doi:10.1186/s12955-016-0500-0) contains supplementary material, which is available to authorized users.

## Background

In the last few decades, it has been recognised that it is imperative to include health-related quality of life (HRQoL) as an outcome measure in the evaluation of treatment interventions in patients with chronic obstructive pulmonary disease (COPD) [1, 2]. COPD is a chronic respiratory condition that cannot be cured; therefore, many COPD treatment programmes focus on the self-management of symptoms and their effect on the patient’s HRQoL [3].

Currently, HRQoL in patients with COPD is typically measured by means of standardised self-report questionnaires that were developed using Classical Test Theory (CTT) [4]. Although most HRQoL questionnaires have been extensively validated, their use is not without limitations; many of these limitations stem directly from the static nature of the current generation of questionnaires [5]. To facilitate the comparison of scores within and among patients, the same questions need to be administered to each patient at each time-point. This means that a single set of questions should be suitable to assess the entire underlying range of HRQoL (from very good to very poor) and should provide sufficient measurement precision at all levels in between. Consequently, a large number of questions are typically required to achieve both sufficient measurement width (content validity) and precision (reliability). This places a considerable burden on patients, who have to complete numerous items, many of which seem irrelevant or redundant to their specific situation. Ideally, each questionnaire should be tailored to the individual patient, resulting in each item (question) soliciting valuable information. However, this should not result in a lack of comparability across patients. This flexibility can be achieved using modern techniques: computerised adaptive testing (CAT). CAT [6] is a specific type of computer based testing that uses an Item Response Theory (IRT) [7] measurement model for item selection during test taking. IRT and CAT were first used in the field of educational measurement. In the last few decades, both techniques have become increasingly popular in health research. Item selection in a CAT is dependent on a patient’s estimated score on one or more latent traits. The estimate of the score on the latent trait (here: HRQoL) of the patient is continuously adjusted (each time an answer to an additional item is given) until a specific pre-defined criterion is reached [8]. This procedure permits a higher degree of precision with fewer items than a procedure using static scales [8]. CAT is scored in real-time; results can be displayed to the physician and/or patient almost instantly in written and graphic reports.

A CAT selects items from a pool of items: an item bank. An item bank ideally consists of a large number of items covering all relevant aspects of the construct under study. An item bank can be developed from scratch, or built on the foundations of previous work (e.g., using items from existing questionnaires as a starting point) [5, 8]. Item bank development usually includes both quantitative and qualitative methods; i.e., respectively, evaluating the item performance using an IRT model, and conducting cognitive interviews or focus groups in order to obtain in-depth understanding of the way the construct is perceived by members of the target population and cognitive interviews to improve item formulation (see e.g., [9–15]). It is paramount that the items be of good quality, both in terms of content validity and psychometric properties: a CAT can only be as good as the item bank it is based on [8]. After the key concepts to be included in the bank have been identified, the formulation and presentation of the items has been found adequate, and the psychometric properties of the items favourable (acceptable coverage of latent trait values, adequate measurement precision where it is needed) a final calibration of the item bank is performed. From this point onward the item parameters are considered “known” and can be used for item selection in CAT.

There is a need for flexible, accurate, and efficient assessment of quality of life in COPD. Currently, there is no gold standard. The SGRQ and SGRQ-C are two of the best-known legacy measures and have been shown to be of high quality; however, they might be viewed as problematic or unsuitable for use in (routine) practice, due to their length. The purpose of this paper is to describe the development of the COPD-SIB: a COPD-specific HRQoL item bank that can be used to inform CAT, covering topics that are relevant to COPD patients. We report on both qualitative (item selection and generation) and quantitative (psychometric analysis using IRT) aspects of this process.

## Methods

### Item selection and development

**Fig. 1:**
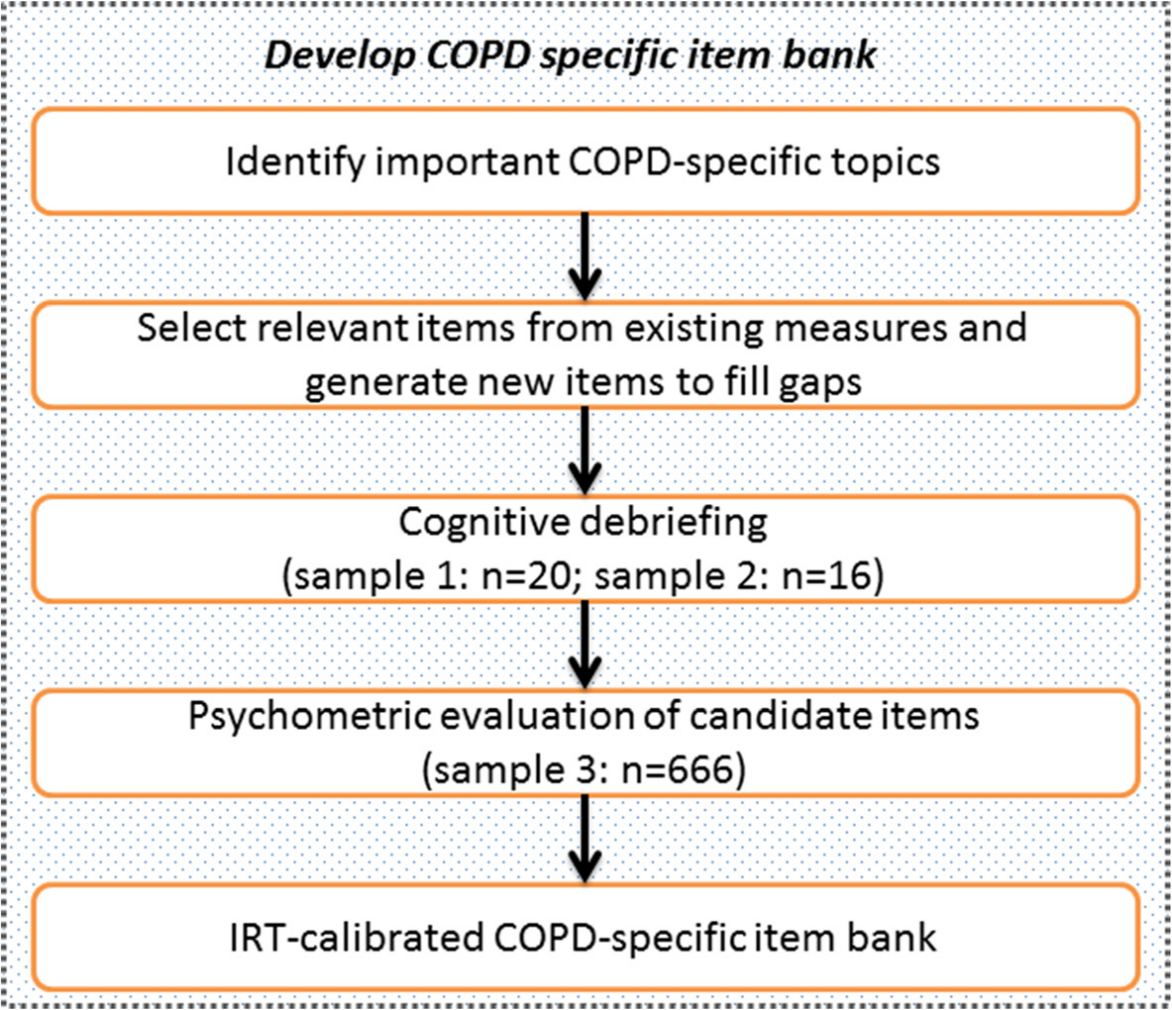
Flowchart of the development process of the COPD-specific item bank (COPD-SIB)

A predefined structured item generation methodology was used to select and design items for the COPD-SIB. This procedure consisted of three steps (which are illustrated in Fig. 1). First, it was determined which topics should be covered. Topics were identified by conducting a literature review and by re-analysing interviews with patients conducted previously [9]. This task was performed by LL under the supervision of MP. Second, relevant items were selected from existing instruments based on the findings of step 1, and new items were written to fill gaps (defined as topics that were not sufficiently covered). This task was jointly performed by LL and MP, and reviewed by JP. Third, the items selected and developed in step two were evaluated for relevance and clarity in several sets of cognitive interviews (see Additional files 1 and 2); the results from these interviews were used to further improve the items and fill newly identified gaps (defined as topics that had not been identified in a previous step but emerged as highly relevant based on the interviews conducted in step 3). This task was primarily performed by MP, with contributions from LL and under the supervision of JP.

The St. George Respiratory Questionnaire for COPD patients (SGRQ-C) was taken as a starting point, since it is widely used and contains many items of high quality [16, 17]. Items from other instruments were considered for inclusion if a) they pertained to themes considered important by COPD patients (importance was deduced from interviews and literature review), and b) they did not show too much overlap with SGRQ-C items. Permission from the developers of the questionnaire for use of these items was a requirement. We included items from five existing questionnaires in our initial item pool: the SGRQ-C, the Quality of Life for Respiratory Illness Questionnaire (QoL-RIQ), the COPD Assessment Test, the Maugeri Respiratory Failure Questionnaire Reduced Form (MRF26), and the VQ11 [18–22]. After items had been selected from existing instruments, the topics covered by these items were compared to the ones most frequently mentioned in the patient interviews. Gaps were identified, and new items were written using statements made by patients as a starting point.

For the SGRQ-C and the COPD Assessment Test, official Dutch translations were available. The items selected from the QoL-RIQ, MRF26, and VQ11 were translated into Dutch by an expert; a native Dutch speaker who holds a university degree in English Language and Culture and has ample experience in English-Dutch and Dutch-English translation. She also translated all newly developed Dutch items into English.

All items in the initial item pool were subjected to cognitive debriefing, using the Three Step Test Interview (TSTI) [23]. In this study, only the Dutch items underwent the process of cognitive debriefing and validation. We plan to repeat this process for the English items in a future study, in collaboration with colleagues from Canada [24]. See Additional file 1 for a detailed explanation of this procedure along with example probes.

### Patients

Data from three Dutch COPD patient samples were used for the analyses (see Fig. 1). Purposive sampling was used for samples 1 and 2 (interview data); inclusion stopped when saturation was reached. The inclusion criteria were: a medical diagnosis of COPD; sufficient mastery of the Dutch language; being able to answer questions in a face-to-face interview (samples 1 and 2); being able to complete a questionnaire (samples 1-3). All patients in samples 1 and 2 were recruited through pulmonary clinics in the Netherlands. The patients in sample 3 (questionnaire data) were recruited through healthcare professionals in JP’s professional network. See Additional file 2 for detailed information about the samples.

### Psychometric evaluation of the item bank

#### Test design

**Fig. 2:**
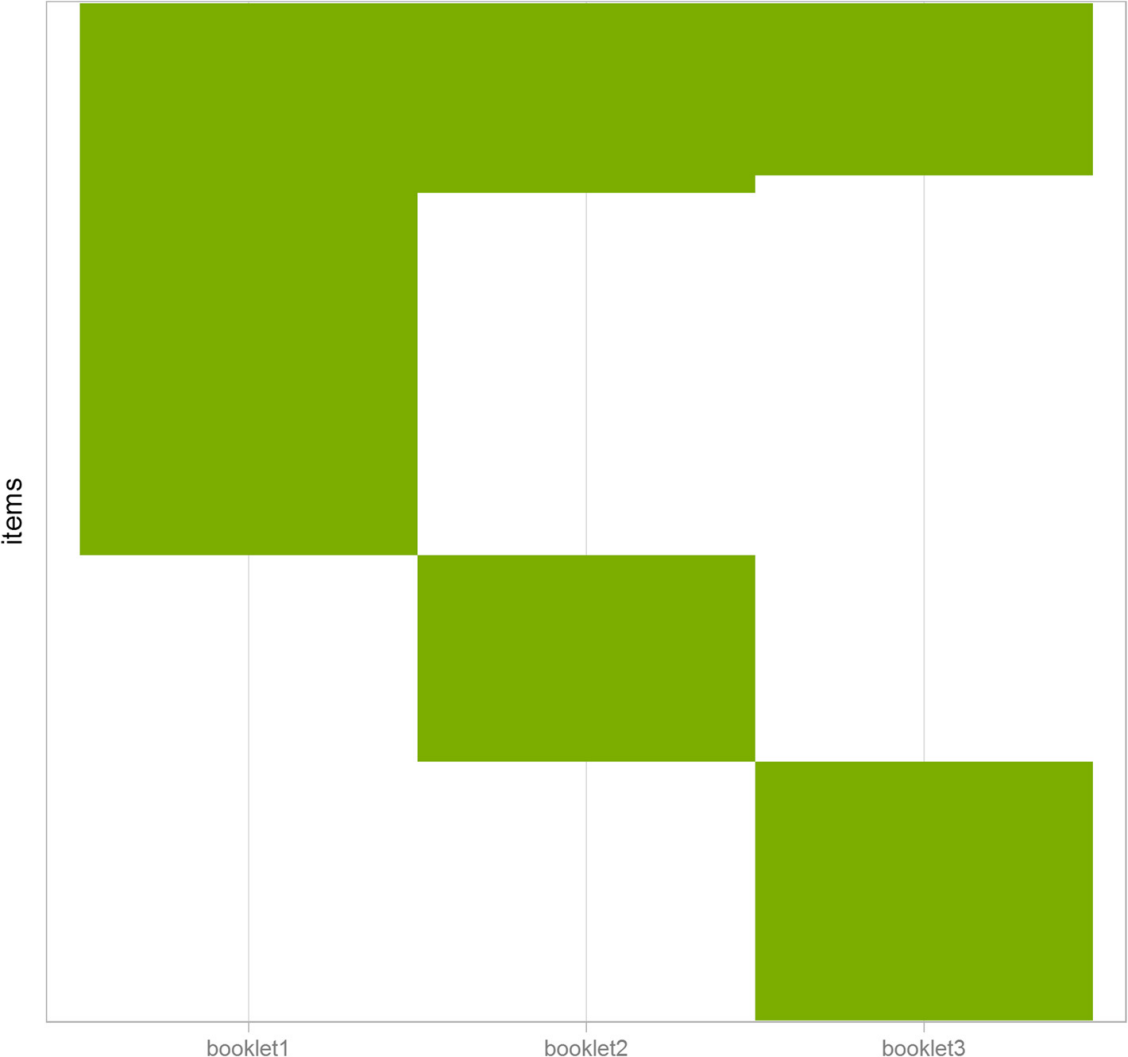
Visual representation of booklet design with number of items on the y-axis and booklet number on the x-axis. Note that the items are ordered according to their booklet assignment to illustrate the design

In addition to evaluating the psychometric properties of the COPD-SIB items, we wanted to establish the measurement properties of three generic HRQoL domains in a Dutch COPD sample. The results for these three domains will be presented in a separate paper. We did not want to create one long questionnaire including all four domains, since this would be very burdensome for patients; therefore we decided to divide the total number of items^1^ over three so-called booklets (questionnaire versions), each containing around 100 items.^2^ The booklets contained between 23 and 32 COPD-SIB items each, of which 10 were anchor items. Anchor items are items that are present in every booklet and which are thought to have stable measurement properties. They can be used to link the items in the different booklets to form a common scale, when using parametric IRT (this procedure is also known as equating) [25]. A widely used guideline to selecting anchor items is that this item set should be a mini-version of the whole item bank, implying that the anchor set should cover the same content (but with fewer items) as the total item bank [25]. The anchor item set used in this study was selected by a content expert (JP) to ensure it adequately reflected the original spread in topics. The other COPD-SIB items were divided randomly over the three booklets. See Fig. 2 for a visual impression of the booklet design, and Table 2 for more information regarding which item was included in which booklet.

#### Assessing item quality and calibrating the item bank

The main purpose of the current study was to develop a unidimensional disease-specific item bank: the COPD-SIB. We wanted to retain only items of sufficient psychometric quality. The Graded Response Model (GRM; an IRT model suitable for Likert scale data) [26, 27] was estimated to obtain item parameters needed for the CAT. Several item fit statistics are currently available for the GRM, such as the *S-X*^*2*^; however, these only have adequate power in very large samples [28]. Unsurprisingly, this statistic did not flag any item for misfit in our analysis. Rather than relying on these outcomes, we used two complementary procedures providing outcomes that are not dependent on the IRT model under evaluation: Mokken Scale Analysis (MSA) [29, 30] and parametric smoothed regression lines based on a generalised additive model (GAM) [31]. MSA was used to identify items that formed a strong unidimensional scale. Items that were flagged for removal by the MSA were further evaluated by visually inspecting the response curves estimated using GAM plots to determine the nature of the misfit. A GAM model is a generalised linear model based on a set of smooth functions; the model does not require a detailed specification of parametric relationships, thus allowing for relatively flexible modelling of statistical relationships (typically involving regression splines) [32].

MSA was performed using the R [33] package Mokken [34]. The model used was the monotone homogeneity model (MHM), which is a nonparametric IRT model. In recent years, MSA has been increasing in popularity in health research (e.g., [16, 35–42]). MSA is a scaling method that identifies scales that allow an ordering of individuals on an underlying one-dimensional scale using the unweighted sum of item scores. In order to establish which items co-vary and form a scale, scalability coefficients are calculated on three levels: item-pairs (*H*_*ij*_), items (*H*_*i*_), and scale (*H*). *H* is based on *H*_*i*_ and reflects the degree to which the scale can be used to reliably order persons on the latent trait using their sum score. Similar to the item-rest correlation, *H* also expresses the degree to which an item is related to other items in the scale. A scale is considered acceptable if 0.3 ≤ *H* < 0.4, good if 0.4 ≤ *H* < 0.5, and strong if *H* ≥ 0.5 [12; 13].

The MSA analyses were performed for each booklet separately (since MSA cannot account for the type of test design we used). We first performed confirmatory analyses, using *H* ≥ 0.3 as a cut-point for an acceptable scale. Since the *H*-value for one of the booklets fell below the cut-point, the confirmatory analyses were followed by exploratory analyses, again using *H* ≥ 0.3 as a cut-off. In an exploratory MSA scales are formed in an iterative manner; the selection algorithm starts with two good items, adding one item at a time using certain criteria (*H*_*i*_ ≥ user-specified cut-off; the item under consideration does shows a positive relationship in terms of *H*_*ij*_ with other items in the scale). Two selection algorithms are currently available; we chose to use the newer one, the genetic algorithm [43].

The GRM was fitted and parameters were estimated using the R package mirt [44]. Metropolis-Hastings Robbins-Monro (MH-RM) estimation was used with a tolerance threshold of 0.001. The algorithm converged after 602 iterations. The GAM plots were also produced using the mirt package (function itemGAM).

## Results

### Item selection and development

#### Domain definition

Eight important themes not covered by PROMIS domains were identified based on literature review and patient interviews:

1. Coping with disease/symptoms, adaptability
2. Autonomy
3. Anxiety about the course/end-state of the disease, hopelessness
4. Positive psychological functioning
5. Situations triggering or enhancing breathing problems
6. Symptoms
7. Activity
8. Impact

Items that pertained to these eight themes were selected/written to be included in the COPD-SIB item bank.

#### Item generation and revision

The items that were selected for psychometric evaluation are listed in Table 1 (English version). Note that the items were coded in such a way that a higher score on the latent trait is indicative of better quality of life. We decided not to include the COPD Assessment Test items, since patients were confused by the format (most patients only read/paid attention to the left half of the items). The SGRQ-C items, on the other hand, were generally very well-received by patients. We used the findings reported by Paap et al. [17] to inform item revision for the SGRQ-C items that were included in the initial item pool.

**Table 1:** Overview over items selected for psychometric evaluation

Item nr	Source^a^	Original Item	Revised Item	Revised response format (RF) and instruction (In)
1	QoL-RIQ	Being in air-conditioned buildings	Being in air-conditioned buildings (for instance, in hospitals)	RF1, In1
2	QoL-RIQ	On cold days		RF1, In1
3	QoL-RIQ	On foggy days		RF1, In1
4	QoL-RIQ	On humid days		RF1, In1
5		On windy days		RF1, In1
6	QoL-RIQ	Being outside during the polling season		RF1, In1
7	QoL-RIQ	Due to domestic animals or pets		RF1, In1
8	QoL-RIQ	By flowers, trees, plants		RF1, In1
9	VQ11	I feel unable to achieve my objectives	Because of my COPD, I feel unable to achieve all of my objectives.	RF2, In2
10		I am confident I will be able to cope with my COPD, even if the complaints get worse.		RF2^b^, In2
11		I can imagine that there are people with severe COPD complaints, who feel that life is not worth living anymore.		RF2, In2
12		I don’t like having to ask somebody to help me, when I cannot do something myself.		RF2, In2
13		Because of my COPD, I appreciate my friends more.	Because of my COPD, I appreciate my social contacts (e.g., friends, partner, relatives) more.	RF2^b^, In2
14		When I think about my COPD, I have a feeling of hopelessness.		RF2, In2
15		I shun activities I know will cause fatigue and breathlessness.	I shun activities I know will cause fatigue.	RF2, In2
16		Since being diagnosed with COPD, I have lived more consciously.		RF2^b^, In2
17		I find it frustrating that I have to accept help for things I was used to doing myself.		RF2, In2
18		If my COPD symptoms get worse, I don’t care about life anymore.		RF2, In2
19		I am content with the things I can still do.		RF2^b^, In2
20		I feel disappointed, when I’m not able to do something because of my COPD.		RF2, In2
21		Because of my COPD I’m afraid of being alone.		RF2, In2
22		When I worry about my COPD, I find it hard to talk about it.		RF2, In2
23		I don’t feel restricted, due to my COPD.	I feel restricted, due to my COPD.	RF2, In2
24		I value my life just as much as I did before I was diagnosed with COPD.		RF2^b^, In2
25		I shun activities I know will cause fatigue and breathlessness.	I shun activities I know will cause breathlessness.	RF2, In2
26		I avoid thinking about how my COPD could get worse in the future.		RF2, In2
27		Once in a while I have such shortness of breath that I fear I will suffocate.	Once in a while I have such shortness of breath/wheezy chest that I fear I will suffocate.	RF2, In2
28		I find it very hard to accept that I cannot do everything I would like to do, due to my COPD.	I find it hard to accept that I cannot do everything I would like to do, due to my COPD.	RF2, In2
29	QoL-RIQ	Feeling dependent upon others	I don’t like the feeling of being dependent upon others.	RF2, In2
30	MRF-26	Because of my lung disease, I cannot talk as much as I would like to.	Because of my COPD, I cannot talk as much as I would like to.	RF2, In2
31		Because of my COPD, I am sometimes unable to control my bowel movements.		RF2, In2
32	MRF-26	Because of my COPD, I visit friends and acquaintances less frequently than I used to.	Because of my COPD, I go out to see friends or acquaintances less than usual.	RF2, In2
33	MRF-26	Because of my COPD, I spend much more time alone.	Because of my COPD, I spend much more time alone.	RF2, In2
34	MRF-26	Because of my COPD, I would like somebody to accompany me, when I go out	Because of my COPD, when I am outside I feel I need to have someone with me.	RF2, In2
35	SGRQ-C	My cough hurts		RF2, In2
36	SGRQ-C	My cough makes me tired		RF2, In2
37	SGRQ-C	I am breathless when I talk	I get breathless when I talk	RF2, In2
38	SGRQ-C	I am breathless when I bend over	I get breathless when I bend over	RF2, In2
39	SGRQ-C	My cough or breathing disturbs my sleep		RF2, In2
40	SGRQ-C	I get exhausted easily	I get tired easily	RF2, In2
41	SGRQ-C	My cough or breathing is embarrassing in public	I feel ashamed when I have to cough or when I have difficulty breathing in the presence of other people	RF2, In2
42	SGRQ-C	My chest trouble is a nuisance to my family, friends or neighbours	I feel that my chest trouble is a nuisance to my environment (e.g. family, friends or neighbours)	RF2, In2
43	SGRQ-C	I get afraid or panic when I cannot get my breath		RF2, In2
44	SGRQ-C	I feel that I am not in control of my chest problem	I have the feeling that I am not in control of my chest problem	RF2, In2
45	SGRQ-C	I have become frail or an invalid because of my chest	I have become frail or an invalid because of my chest problem	RF2, In2
46	SGRQ-C	Everything seems too much of an effort		RF2, In2
47	SGRQ-C	My breathing makes it difficult to do things such as walk up hills, carrying things up stairs, light gardening such as weeding, dance, play bowls or play golf	My breathing problems make it difficult to do light gardening, such as weeding.	RF2, In2
48	SGRQ-C	My breathing makes it difficult to do things such as carry heavy loads, dig the garden or shovel snow, jog or walk at 5 miles per hour, play tennis or swim.	My breathing problems make it difficult to exercise (e.g., jogging, playing tennis, or swimming).	RF2, In2
49	SGRQ-C	My breathing makes it difficult to do things such as walk up hills, carrying things up stairs, light gardening such as weeding, dance, play bowls or play golf.	My breathing problems make it difficult to do things such as dancing, playing golf, or playing bowls.	RF2, In2
50		It frustrated me that I couldn’t do everything I wanted to do anymore.		RF3, In3
51		I thought sometimes, I’m really fed up with everything.		RF3, In3
52		I wanted to stay in bed/lie down on the couch all day, when I had a “bad” day.		RF3, In3
53		I resigned myself to the fact that I was not able to do certain things anymore, due to my COPD.	I could accept it, when I was not able to do something anymore, due to my COPD.	RF3^b^, In3
54		I tried to find an alternative when I could not perform a certain activity due to my COPD.	I persevered until I had finished an activity, despite the fact that I couldn’t perform that activity well, due to my COPD.	RF3^b^, In3
55		I panicked, when I had trouble breathing		RF3, In3
56		I could cope with my COPD.		RF3^b^, In3
57		I got my breathing problems under control.		RF3^b^, In3
58	SGRQ-C	I cough	I coughed.	RF3, In3
59	SGRQ-C	I bring up phlegm (sputum)	I brought up phlegm (sputum).	RF3, In3
60	SGRQ-C	I have shortness of breath	I had shortness of breath.	RF3, In3
61	SGRQ-C	I have attacks of wheezing	I had attacks of wheezing.	RF3, In3
62	SGRQ-C	Getting washed or dressed		RF3, In4
63	SGRQ-C	Walking around the home		RF3, In4
64	SGRQ-C	Walking outside on the level	Going for a walk	RF3, In4
65	SGRQ-C	Walking up a flight of stairs	Walking up a flight of stairs (one floor)	RF3, In4
66	SGRQ-C	Walking up hills	Walking up a steep hill	RF3, In4

RF1: 4 = Not at all; 3 = A little bit; 2 = Somewhat; 1 = Quite a bit; 0 = Very much

RF2: 4 = Strongly disagree; 3 = Disagree; 2 = Neither agree nor disagree; 1 = Agree; 0 = Strongly agree

RF3: 4 = Never; 3 = Rarely; 2 = Sometimes; 1 = Often; 0 = Always

In1 = “How much have you been troubled by breathing problems due to the following circumstance?”

In2 = “Please, indicate the degree to which you agree or disagree with the following statement”

In3 = “In the past 7 days…”

In4 = “Please, indicate whether the following activity causes shortness of breath. If the weather influences your complaints, assume the weather conditions are favourable, when you answer this question”

^a^If the source is not given, it concerns a newly written item

^b^The item scores for these items need to be reversed prior to analysis due to positive wording

We followed an iterative procedure (three revision rounds) for the remaining items, since this subset of the item pool included newly written items. Patients clearly had trouble switching back and forth between different response formats, and strongly objected to dichotomous response options. Therefore, we decided to standardise the response format for all items in the item bank to a 5-point Likert-scale reflecting magnitude (“not at all” to “very much”), frequency (“never” to “always”), and agreement (“strongly disagree” to “strongly agree”). Composite items were split into separate ones, double negations were rephrased, and the expression “lung disease” was changed to “COPD”. See Table 1 for the original and revised item texts.

### Preparing the data for psychometric analysis

A large number of items had low endorsement (n < 10) for at least one response option/category. This can cause problems in psychometric analyses; hence, the problematic categories were merged with adjacent categories for these items. Note that items having different numbers of response categories due to merging does not constitute a problem for the GRM, nor does it hamper the comparison of item discrimination parameters (estimated with the GRM) among items. See Additional file 3 for the R code used to merge item categories. Three items were removed at this stage, due to a large number of missing values (>20 %) per booklet: items 6, 7 and 8.

### Psychometric evaluation of the item bank

#### Assessing item quality: results of the MSA and visual inspection of GAM plots

MSA requires a complete data-set. Therefore the MSA analysis was repeated for each booklet separately and two-way imputation was used to create a complete data-set for each booklet (2-4 % missing values per booklet) [45, 46].

The confirmatory analyses resulted in acceptable *H-*values for booklets 1 (.30) and 3 (.31), but a low *H*-value for booklet 2 (.26). Taking the results of the three exploratory MSA’s together, 19 items (see Table 2) were flagged as problematic (most of them had very low or even negative *H*_*ij*_ values and were not assigned to any scale). If these items would have been excluded from the analyses, the *H*-values would have equalled .43, .40, and .43 for booklets 1, 2, and 3, respectively.

**Table 2:** Item properties

Item nr	item content (key words)	$\widehat{\boldsymbol{\alpha}}$	$\widehat{{\boldsymbol{\beta}}_1}$	$\widehat{{\boldsymbol{\beta}}_2}$	$\widehat{{\boldsymbol{\beta}}_3}$	$\widehat{{\boldsymbol{\beta}}_4}$	booklet	flagged in MSA (booklet)
1	air-conditioning	1.06	−2.64	−1.08	0.27		1	
2	cold	1.15	−3.1	−0.92	0.32	1.54	1, 2, 3	
3	fog	1.4	−2.08	−0.28	0.71	1.83	1	
4	humidity	1.39	−2.32	−0.33	0.77	1.64	1	
5	wind	1.25	−2.09	−0.38	0.62	1.88	1, 2	
9	achieving objectives	1.8	−1.04	0.83	1.38	2.31	1, 2, 3	
10	confidence	0.31	−11.43	−5.73	−2.02	7.99	1, 2, 3	1, 2, 3
11	life worth living	0.76	−2.28	0.56	2.2	3.72	2	2
12	asking for help	0.81	−1.78	1.39	2.24	4.16	1, 2, 3	2, 3
13	friendship	−0.59	4.97	2.5	0.57	−3.6	3	3
14	hopelessness	1.69	−2.51	−0.84	0.15	1.34	1	
15	fatigue	1.21	−2.27	0.01	0.94	2.7	1, 2, 3	
16	living consciously	−0.52	5.69	2.59	0.23	−5.77	2	2
17	accepting help	1.52	−1.05	0.72	1.46	2.29	1	
18	life worth living 2	1.32	−2.71	−1.57	−0.38	1.18	3	
19	feeling content	0.26	−11.08	−6.75	−3.34	4.8	1	1
20	feeling disappointed	1.35	−2.06	−0.4	0.68	2.47	2	
21	fear of being alone	1.69	−1.65	−0.44	0.75		2	
22	talking about anxiety	1.1	−1.02	−0.04	1.85		1	
23	feeling restricted	2.33	−1.08	0.5	1.16	1.87	3	
24	valuing life	0.71	−4.84	−2.22	−0.93	2.64	3	3
25	shunning activities	1.43	−2.28	0.16	0.85	2.26	2	
26	avoidance	0.65	−4.09	−0.44	1.17	3.68	1	1
27	fear of suffocation	1.94	−2.18	−0.87	−0.14	1.12	1, 2, 3	
28	not accepting restrictions	1.61	−1.32	0.56	1.31	2.41	1	
29	dependence	2.08	−2.12	−0.44	0.03	1.2	2	
30	talking	1.72	−2.34	−0.65	−0.06	1.22	3	
31	bowel problems	0.86	−2.91	−1.18	0.66		1	1
32	friendship 2	1.91	−2.06	−0.75	−0.18	0.94	1, 2, 3	
33	alone	1.74	−2.1	−0.75	−0.13	1.18	3	
34	fear of being alone 2	1.67	−1.39	−0.63	0.95		2	
35	cough hurts	1.41	−1.68	−0.5	0.98		1	
36	cough tired	1.79	−1.78	−0.05	0.58	1.43	1	
37	breathless talk	1.48	−0.53	0.24	1.69		2	
38	breathless bend	1.52	−1.87	0.07	0.72	1.9	3	
39	sleep disturbed	1.44	−2.63	−0.75	0.09	1.54	1, 2, 3	2
40	exhausted	2.04	−1.13	0.96	1.43		3	
41	cough embarrassing	1.11	−3.35	−1.19	−0.18	1.94	1, 2, 3	3
42	nuisance to others	1.97	−2.21	−0.86	−0.15	1.22	2	
43	panic	2.4	−1.86	−0.63	0.04	1.19	2	
44	not in control	1.64	−1.41	−0.39	1.59		1	
45	frail, invalid	2.33	−1.75	−0.28	0.3	1.28	2	
46	effort	2.22	−1.99	−0.56	0.16	1.54	3	
47	activities (gardening)	2.34	−1.13	0.11	0.62	1.64	1	
48	activities (exercise)	1.55	−0.82	0.92	1.54		3	3
49	activities (dancing)	2.12	−0.88	0.35	0.81	1.8	1	
50	frustration	2.21	−2.07	−0.7	0.46	1.2	3	
51	being fed up	1.7	−2.41	−1.37	−0.66		1	
52	wanting to stay in bed	1.35	−2.14	−0.94	0		3	
53	acceptance	−0.32	4.93	2.4	−2.92	−8.17	2	2
54	adapting	0.12	−14.8	−7.53	3.94	20.35	3	3
55	panic 2	1.88	−2.06	−0.88	0.25		2	
56	coping	1.08	−3.1	−1.08	0.97		3	
57	control	1.39	−1.05	0.74			1	
58	cough	0.74	−3.97	−0.92	1.51	3.32	1	1
59	phlegm	0.8	−3.27	−1.43	0.52	2.17	1	1
60	short of breath	1.95	−1.58	−0.22	1.55		1	
61	wheezing	0.81	−3.47	−1.51	0.49	2.02	1	1
62	breathless wash	2.28	−1.47	−0.64	0.1	0.66	3	
63	breathless walk 1	2.49	−2.07	−1.25	−0.2	0.47	3	
64	breathless walk 2	0.98	−1.98	−0.51	1.24	2.31	1, 2, 3	1, 2
65	breathless stairs	0.77	−1.52	−0.19	1.35	2.92	2	2
66	breathless hills	0.04	−17.48	6.54	22.32	36.14	1	1, 2

*Note*: the reported parameter estimates were calculated using the GRM; the last two columns indicate in which booklet the item was included, and whether or not the item was flagged for removal in the Mokken Scale Analysis (MSA)

**Fig. 3:**
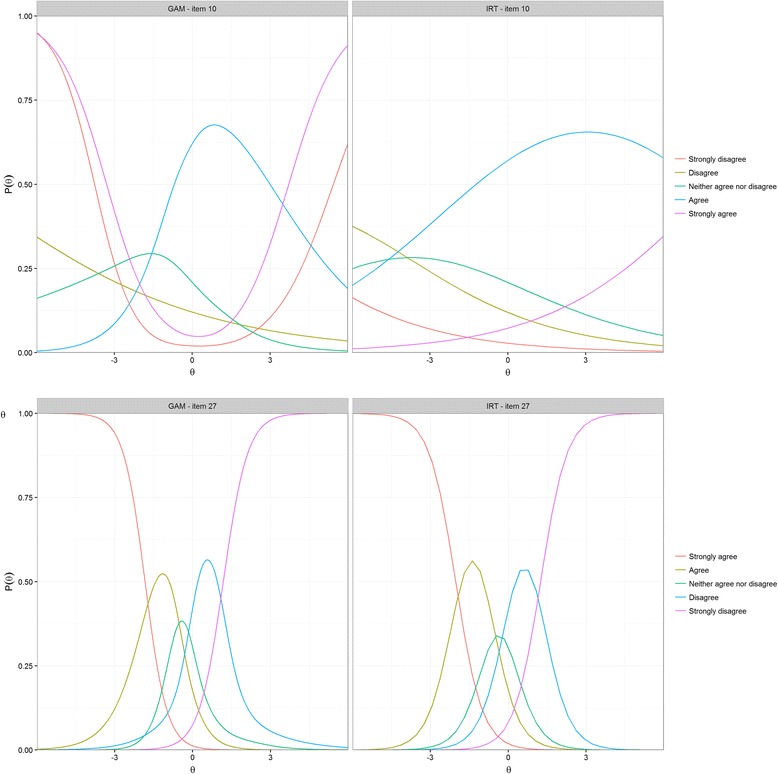
Option response curves as estimated using the GRM (on the right), and parametric smoothed regression lines based on a GAM (on the left) for an item with good fit to the GRM (item 27), and one with bad fit (item 10)

Visual inspection of the GAM plots (smoothed regression lines) for the items flagged for removal in the MSA revealed substantial differences between one or more response curves as estimated under the GAM as compared to their counterparts estimated under the GRM, for most items. In some cases, one or more of the response curves was hard to estimate (very erratic, with multiple peaks). For five items (10, 19, 24, 53, 54), a very striking type of misfit was identified: the GAM plots showed that one or more response curves were U-shaped, indicating that both patients with very high and very low θ-scores scores were likely to endorse these response categories (see Fig. 3 for example plots).

#### Calibrating the item bank: results of the parametric IRT analysis

Table 2 shows the estimated parameters based on the GRM for 63 out of 66 items.^3^ Up to five parameters are calculated in this model: the slope (denoted *α*) and the thresholds (denoted *β*_*j*_). The slope of an item expresses its ability to discriminate among persons with low and high HRQoL; it is also indicative of how strongly this item is related to the latent trait (denoted θ). The threshold parameters indicate the point on the latent trait scale at which 50 % of the patients would choose the response category in question or higher. Since the probability is always 100 % for choosing the lowest category or higher, there is no threshold for the lowest category. Originally, all items were scored on a 5-point Likert scale ranging 0-4; however, since we had to collapse some response categories due to data sparseness, not all items in Table 2 have four thresholds. For example, for item 21 (“Because of my COPD I’m afraid of being alone.”), the categories 0 (strongly agree) and 1 (agree) were merged. Thus, the probability of choosing at most *neither agree nor disagree* is 50 % for patients with a θ-score of -2.79; the probability of choosing at most *disagree* is 50 % for patients with a θ-score of -0.736; and the probability of choosing *strongly disagree* is 50 % for patients with a θ-score of 1.267.

**Fig. 4:**
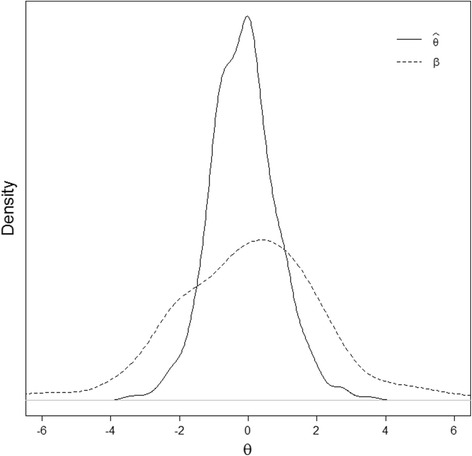
Distribution of estimated theta-values (solid line) and of estimated beta parameters (dashed line); both estimated using the Graded Response Model

The metric of the threshold values is determined by the distribution of θ. A standard normal distribution (mean = 0, SD = 1) was assumed when estimating the model (this is done to identify the model, similar to confirmatory factor analysis; in Bayesian terms this can be considered as a prior distribution). The threshold values as well as θ-scores may be interpreted relative to this distribution. Bayesian expected a-posteriori (EAP) scoring was used to estimate the θ-scores. The EAP estimator uses prior information (in this case the estimated population distribution in the fitted model) in calculating θ-scores. When this method is used, extreme scores are pulled in toward more realistic values. This is especially useful in cases where patients endorse either the lowest or highest response category on all items, in which case the maximum likelihood estimate is undefined. Figure 4 depicts the distribution of estimated θ-scores as well as the estimated threshold parameters. Both distributions look reasonably normal, and the threshold parameters cover the entire range of relevant θ-values (see Fig. 4).

**Fig. 5:**
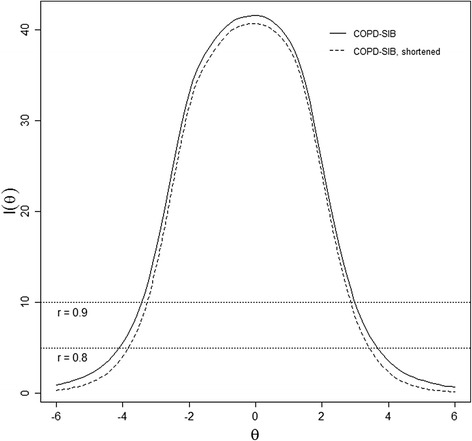
Information Function for the full item bank (solid line) and the shortened item bank (dashed; problematic items removed)

The information function (Fig. 5) shows that the item bank covers all relevant θ-values (>99 % of θ-values fall in the range of -3 and +3). This figure depicts the measurement precision as a function of θ. An information value of 5 corresponds with a reliability of 0.8. The information function is the sum of the item information functions; each item gives most information close to its thresholds, and items with higher slopes give more information.

### Selecting items for the final item bank

As can be seen from Table 2, 17 out of 20 items flagged by the MSA had low (<1) or even negative α values. For three flagged items (39, 41, 48), no clear reason for misfit could be identified (acceptable item parameters, no obvious difference between GAM and GRM plots). These three items were therefore retained in the item bank. The GRM was estimated again after removal of the 17 problematic items. The resulting item parameters can be found in Additional file 4. This set of 46 items can be considered as the final item bank. Removing problematic items did not have a substantial effect on the information function (Fig. 5).

## Discussion

This paper describes the development of an item bank that measures disease-specific quality of life in patients with COPD: the COPD-SIB. We started out with 66 items (including SGRQ-C items) covering content described as highly relevant by patients, healthcare professionals, and the literature. These items were assessed using complementary psychometric techniques and the data of 666 Dutch COPD patients. The final item bank contains 46 items that form a strong scale. This item bank could be used as a stand-alone instrument, either in full-bank form; better yet, it could be used as the basis for CAT.

Seven items stood out among misfitting items: they had negative slope parameters and/or one or more response curves were U-shaped. Negative slope parameters were found for four items (item 13: “Because of my COPD, I appreciate my social contacts (e.g., friends, partner, relatives) more”; item 16: “Since being diagnosed with COPD, I have lived more consciously”; item 53: “I could accept it, when I was not able to do something anymore, due to my COPD”; item 54: “I persevered until I had finished an activity, despite the fact that I couldn’t perform that activity well, due to my COPD”), while U-shaped response curves for one or more categories were found for four items (item 10: “I am confident I will be able to cope with my COPD, even if the complaints get worse”; item 19: “I am content with the things I can still do”; item 24: “I value my life just as much as I did before I was diagnosed with COPD”; and item 53). When comparing the content of these items to other items in the bank, it is apparent that these items are all worded in a positive way whereas most items in the bank are not. Only one positively worded item showed good fit (item 57: “I got my breathing problems under control.”). The reason we included items with a more positive item formulation, was that several patients indicated that they felt it did not do their situation justice if the item bank would only consist of negative items. Patient quotes were used to inform the formulation of these items. Our results illustrate that it can be difficult to optimise content validity while simultaneously maintaining the same level of construct validity (under a given model); in this case, adding items to improve content validity resulted in multidimensionality. It has been previously suggested that including reversed worded items in a questionnaire might affect reliability and aspects of validity [47, 48]. Patients may not notice that some items are formulated in a reversed way, or they might be confused by this reversal in meaning. As an effect, there may be an increase in measurement error and/or a method/artifical second factor may be found in dimensionality analyses caused by response bias [49]. To prevent response bias caused by inattention or confusion, it may be advisable to present positively and negatively worded items separately in a future study, as suggested by Roszkowski and Soven [50]. Another possibility would be to create separte item banks for positively and negatively worded items; PROMIS follows this strategy for a number of domains (e.g., [51, 52]). If these strategies do not solve the issue of U-shaped response curves, it may be worth while to re-analyse the data with a different IRT model, which allows for peaked/dipped response curves (a so-called “unfolding model”) [53].

We developed 29 new items that were subjected to cognitive debriefing along with a selection of items from existing questionnaires. Initially, the answering categories provided for the newly developed items were dichotomous: agree/do not agree. A substantial number of patients indicated that they were unhappy with only having two options, and asked for Likert scales. We made adjustments accordingly, and decided to harmonise the answering categories of all items following PROMIS guidelines. Patients were happy with the 5-point Likert scales. Our findings illustrate, however, that this not necessarily means that patients will use the entire scale. The resulting data sparseness poses challenges when modelling the data. A widely used solution is to merge adjacent categories, which is also what we did for a number of items. This solution is not popular with everyone; but since having a very low cell count for certain item-category combinations leads to problematic parameter estimates (very high or low, large standard error) it is unavoidable in practice. In such cases, it may be advisable to use a model that is unsensitive to differences in the number of categories per item after merging, such as the GRM we used in this study. We suggest that this approach (providing the patient with the response scale of their preference, subsequently merging categories prior to analysis, and finally using an appropriate model) is to be preferred to avoiding dealing with the field of tension between patient perspective and psychometric considerations.

## Conclusions

In the development of the COPD-SIB, the patient perspective has taken a central role. The item bank contains items tapping into several topics described as highly relevant by patients and the literature. We used complementary psychometric techniques to evaluate the candidate items, and the final selection forms a strong unidimensional scale. The COPD-SIB is a promising candidate to measure COPD-specific HRQoL in routine practice; especially when used to build a CAT (time efficient, while not compromising measurement precision). The COPD-SIB was developed using a large Dutch sample of COPD patients. The Dutch version of the item bank is ready for use, and available upon request (contact MP or JP). First steps toward cross-cultural validation are currently underway [9, 24].

## Additional files

Additional file 1: Detailed explanation of Three Step Test Interview (TSTI) along with example probes. (PDF 92 kb) Additional file 2: Detailed description of the samples used in this study. (PDF 121 kb) Additional file 3: R code used to collapse response categories. (PDF 54 kb) Additional file 4: Calibration results of the final version of the COPD-SIB item bank. (PDF 294 kb)

## Footnotes

1 The total number of items equaled 211: 148 for the 3 generic domains and 63 for the COPD-SIB.

2 An informal feasibility study indicated that patients felt that completing up to 100 items was acceptable (n = 4; data not shown).

3 As mentioned previously, three items (items 6, 7 and 8) were excluded prior to analysis since they had >20 % missing values.
