# Supplementary material for: The COPD-SIB: a newly developed disease-specific item bank to measure health-related quality of life in patients with chronic obstructive pulmonary disease
Source: Health Qual Life Outcomes. 2016 Jun 27;14:97. doi: 10.1186/s12955-016-0500-0 (PMC4924274; doi:10.1186/s12955-016-0500-0)
Supplement: Additional file 1: — Detailed explanation of Three Step Test Interview (TSTI) along with example probes. (PDF 92 kb) [file 12955_2016_500_MOESM1_ESM.pdf]

Supplement 1: the TSTI

In the Three Step Test Interview (TSTI) (Hak, Van der Veer, & Jansen, 2008), two important types of cognitive interviewing techniques are combined: think-aloud (step 1) and retrospective verbal probing (step 2 and 3).

During the first step of the TSTI, the interviewer observes the respondent as they are completing the questionnaire and verbalising the thoughts they have while doing so. Among the advantages of the think-aloud technique are "freedom from interviewer-imposed bias" and the "open-ended format"; the patient's responses are not influenced by the interviewer, and may provide information that is unanticipated by the interviewer (Willis, 2005). If necessary, the interviewer gently reminds the patient to think aloud while completing the items. During this phase, the interviewer takes notes about the answering behaviour of the patient (e.g., skipping an item, taking a long time to complete an item, or re-reading the item several times); this information is used as input to formulate probes in step 2.

During step 2 of the TSTI, the interviewer used spontaneous probes (see Table S1 for example probes) based on observations from step 1, which offers flexibility, ensuring the used probes are relevant to the patient, and no suggestive questions are posed to the patient or words are put into their mouth. One of the advantages of probing is that this technique can elicit very specific information of interest to the interviewer. In the case of questionnaire development, it is typically used to assess the clarity of instructions, items, and response choices. In this step the interviewer gives feedback to and shares their observations with the patient in connection with the latter’s behaviour, and invites them to explain the thought process they had during step 1 when completing each item.

In step 3, the interpretation of the items and the thought process experienced by the patient when filling out the questionnaire is put in a broader (social-biographical) context (Bode & Jansen, 2013). They can share their considerations and opinions about the items and the questionnaire as a whole, and how the item/questionnaire content relates to their own (disease) experience. During this phase, patients can make concrete suggestions toward improving existing items, adding new items or removing specific items. See Bode & Jansen (Bode & Jansen, 2013) for a comprehensive summary of the TSTI. Note that we divided the items in sets of 5-6 items per set, and all three steps of the TSTI were performed for each set.

Table S1 Overview of instructions and examples of probes for each phase of the Three Step Test Interview (TSTI)

**Phase**	**Instruction phase**	**Probes**
Think aloud	In order to gain insight into how you interpret the questions and to be able to follow your thought process while you fill in the questionnaire, I would like to ask you to express your thoughts when you complete the questionnaire. You are allowed to think aloud while you are filling in the questionnaire; meanwhile I will observe you and take notes.<br>I will demonstrate how it works: (interviewer thinks aloud while answering the sample question).<br>*During the past seven days I noticed I was stressed. On a scale of 1 to 5, where 1 is strongly disagree and 5 is strongly agree. Stressed, yes I was somewhat stressed, because I had to meet a deadline this week, so I give it a 4.*<br>Are you clear what is expected of you? Then you may now start completing the questionnaire. You are allowed to think aloud while answering the questions.	Please, express your thoughts aloud.<br><br>I can see that you are thinking. Can you express your thoughts aloud?<br><br>What are you thinking right now?<br><br>I can see that you hesitate to answer. Can you express your thoughts or feelings about that aloud?
Interview retrospectively	After a set of 5 to 6 questions, I will interrupt you and discuss with you what I noticed while I observed how you answered the questions.	I noticed that you had to reread the question several times before you answered it. Can you comment on that?<br><br>You hesitated to answer the question. Why was that?<br><br>When you answered the question, you said that the question did not apply to you; yet, you filled in “agree”. Can you comment on that?<br><br>Can you give an example?

		How would you explain this word in your own words?
Semi-structured interview	At present, the questionnaire is still at the development stage. We will use interviews to establish whether the questions are interpreted the way we intended them. We also would like to know what COPD patients think of the questionnaire, and what suggestions for improvements they might have. Your opinion about the questionnaire is very important to us.	What is your opinion about the answer options?<br><br>Does this question apply to your situation?<br><br>What do you think of the way this question is worded?<br><br>What are your suggestions for improvement of this question?<br><br>Are there specific factors or situations that are not mentioned in the questionnaire, but which are, in your opinion, important when you think about the impact COPD has on your quality of life?<br><br>What is your general opinion about the questionnaire?

Analysing the data obtained using the TSTI

Following Paap et al. (2015), we first made a list of all problems and suggestions for improvement raised by patients for each item separately, using a categorical system based on work by Pool et al. (2010) (e.g. composite item) and Liu et al. (2011) (e.g. preference for altering the phrasing of the question). Subsequently, problems and suggestions for improvements were summarised for each item. Finally, problematic items were discussed by the first and second author and consensus was reached about whether and how the alterations of the items were to be made. Alterations were directly based on the input of the patients.

This procedure was followed iteratively (three rounds). During the first round six patients were interviewed and their input was used to inform modification of the items before using them in the next round of interviews (round 2). This was repeated two times more, until patients were satisfied with the items.

References

Bode, C., & Jansen, H. (2013). Examining the Personal Experience of Aging Scale with the Three Step Test Interview. *Methodology, 9*(3), 96-103.

Hak, T., Van der Veer, K., & Jansen, H. A. M. (2008). The Three-Step Test-Interview (TSTI): An observation-based method for pretesting self-completion questionnaires. *Survey Research Methods, 2,* 143-150.

Liu, R., Buffart, L., Kersten, M., Spiering, M., Brug, J., van Mechelen, W., & Chinapaw, M. (2011). Psychometric properties of two physical activity questionnaires, the AQuAA and the PASE, in cancer patients. *BMC Medical Research Methodology, 11*(1), 1-10. doi: 10.1186/1471-2288-11-30

Paap, M. C. S., Lange, L., van der Palen, J., & Bode, C. (2015). Using the Three-Step Test Interview to understand how patients perceive the St. George's Respiratory Questionnaire for COPD patients (SGRQ-C). *Quality of Life Research*, Advance online publication. doi: 10.1007/s11136-015-1192-3

Pool, J. J., Hiralal, S. R., Ostelo, R. W., van der Veer, K., & de Vet, H. C. (2010). Added value of qualitative studies in the development of health related patient reported outcomes such as the Pain Coping and Cognition List in patients with sub-acute neck pain. *Man Ther, 15*(1), 43-47. doi: 10.1016/j.math.2009.05.010

Willis, G. B. (2005). *Cognitive Interviewing: A Tool for Improving Questionnaire Design*. Thousand Oaks, CA: Sage Publications.
