# Supplementary material for: The COPD-SIB: a newly developed disease-specific item bank to measure health-related quality of life in patients with chronic obstructive pulmonary disease
Source: Health Qual Life Outcomes. 2016 Jun 27;14:97. doi: 10.1186/s12955-016-0500-0 (PMC4924274; doi:10.1186/s12955-016-0500-0)
Supplement: Additional file 2: — Detailed description of the samples used in this study. (PDF 121 kb) [file 12955_2016_500_MOESM2_ESM.pdf]

Supplement 2: detailed description of samples

Samples 1 and 2: The items in the initial item pool (step 2) were tested in two samples of COPD patients. The SGRQ-C items were tested in one group of patients, and the remaining items (items taken from other questionnaires as well as newly developed items) in a second group. The results of the cognitive interviews involving the SGRQ-C are reported elsewhere (Paap, Lange, van der Palen, & Bode, 2015). The SGRQ-C sample consisted of 20 COPD patients (13 women; mean age 63.2 (SD =11.4) years) who were all recruited in Enschede from November 2013 through January 2014. Fifty-five percent were inpatients; 35% had GOLD stage II, 15% GOLD stage III, and 50% GOLD stage IV. The remaining items were tested with 16 patients (10 men; mean age 72.2 (SD =5.75) years) recruited from January 2014 through March 2014. Fifty-six percent were recruited in Enschede, 31% in Zwolle, 6% in Eindhoven, and 6% in Meppel; 25% had GOLD stage II, 38% GOLD stage III, and 38% GOLD stage IV.

Sample 3: A large sample was needed to evaluate the psychometric properties of the item bank. JP reached out to HCPs in his professional network (pulmonologists, general practitioners, physiotherapists, and nurse practitioners), some of who in turn reached out to people in their own networks. HCPs willing to participate distributed printed copies of the item banks among the COPD patients attending their clinics from October 2014 through July 2015. Patients could return the questionnaire themselves free of charge, or return it to the clinic. Descriptive statistics can be found in the table below.

Table S2 Descriptive Statistics Sample 3

	Complete dataset<br>N = 666	Booklet 1<br>N = 216	Booklet 2<br>N = 205	Booklet 3<br>N = 245
**Age**				
Mean (SD)	67.2 (10.23)	68.5 (9.74)	66.4 (10.33)	66.7 (10.46)
Missings (%)	2.3	2.3	2.0	2.4
**Gender (%)**				
Male	52.4	51.9	53.2	52.7
Female	45.9	45.8	45.8	46.1
Missings	1.7	2.3	1.5	1.2
**How many years suffering from COPD**				
Mean (SD)	13.5 (13.22)	12.9 (12.05)	15.0 (16.07)	13.2 (13.02)

Missings (%)	10.8	8.3	14.1	9.8
**Marital status (%)**				
Not married/not cohabiting	9.9	9.7	9.9	9.4
Not married/cohabiting	5.6	5.6	6.4	4.9
Married	58.7	62.0	60.1	55.1
Widow/widower	14.3	11.6	14.3	16.7
Divorced	9.2	7.9	7.9	11.4
Missings	2.6	3.2	2.0	2.4
**Highest educational attainment (%)**				
Primary school	15.9	13.0	19.7	15.1
Prevocational education	32.1	30.6	33.5	32.7
Preparatory secondary vocational education/junior general secondary education	17.4	19.0	18.7	15.1
Secondary vocational education	13.5	13.9	13.8	13.1
Senior general secondary school/pre-university education	5.0	5.1	3.4	6.1
Higher professional education	11.0	12.5	8.4	11.8
University education	2.9	3.2	1.5	3.7
Missings	2.3	2.8	1.5	2.4
**Current situation (%)**				
Full-time employment	8.7	8.8	7.4	9.8
Part-time employment	6.9	7.9	6.9	6.1
Homemaker	9.8	8.8	11.3	9.0
Unemployed	3.5	2.8	3.4	4.1
Disabled	13.8	13.0	14.8	13.9
Retired	55.3	56.0	55.2	55.1
Missings	2.1	2.8	1.5	2.0
**Children (%)**				
No	15.6	16.2	15.3	15.1
Only children living away from home	71.2	73.6	72.9	68.2

One or more children living at home	11.3	7.9	10.3	15.1
Missings	2.0	2.3	2.0	1.6
**Mother tongue (%)**				
Dutch	96.8	97.7	96.6	96.7
Moroccan/Turkish/Arabic	.8	.5	1.0	.8
German/French/English	.9		1.0	1.6
Missings	1.1	1.9	.5	.8
**Sports (frequency) (%)**				
I do not participate in sports (anymore)	46.1	43.5	44.3	49.8
A few times a month	2.0	3.7	1.0	1.2
1-2 times a week	30.6	31.9	34.0	26.9
Appr. 2-3 times a week	15.9	15.3	16.3	16.3
More than 3 times a week	3.6	2.8	4.4	3.7
Missings	1.8	2.8	.5	2.0
**Cigarettes (per day) (%)**				
None	73.9	75.9	78.8	68.2
Less than 5 cigarettes	5.9	5.6	4.9	6.9
5-10 cigarettes	8.0	4.6	9.9	9.4
11-20 cigarettes	7.4	7.4	3.4	10.6
21-30 cigarettes	2.7	4.2	2.0	2.0
31- 40 cigarettes	.3		.5	.4
More than 40 cigarettes	.2		.5	
Missings	1.8	2.3	.5	2.4

References

Paap, M. C. S., Lange, L., van der Palen, J., & Bode, C. (2015). Using the Three-Step Test Interview to understand how patients perceive the St. George's Respiratory Questionnaire for COPD patients (SGRQ-C). *Quality of Life Research*, Advance online publication. doi: 10.1007/s11136-015-1192-3
