# Supplementary material for: The COPD-SIB: a newly developed disease-specific item bank to measure health-related quality of life in patients with chronic obstructive pulmonary disease
Source: Health Qual Life Outcomes. 2016 Jun 27;14:97. doi: 10.1186/s12955-016-0500-0 (PMC4924274; doi:10.1186/s12955-016-0500-0)
Supplement: Additional file 3: — R code used to collapse response categories. (PDF 54 kb) [file 12955_2016_500_MOESM3_ESM.pdf]

Supplement 3: Collapsing response categories

Response categories were collapsed in a two-step procedure. In the first step response categories were assigned a one-to-one mapping to a new set of response categories, based on the number of endorsements for each response category. The following R code was used for this purpose;

```
# Create Mapping
# takes a matrix (or any 2d object) and collapses categories to where each
category has at least threshold responses.
# rather naïve, will collapse to 1 category without warning if data requires.
#
# returns a list of (named) lists, each with two elements - the old and new
response category indices.
# these can easily be used to recode, for example with recode() from the "car"
package.
#
# Author: Karel A. Kroeze <k.a.kroeze@utwente.nl>

# Start from the right, and collapse to the left whenever count < threshold
# Finally, collapse once more if first element is also too low.
create_mapping <- function(data, threshold) {
  counts <- list()
  for (i in 1:ncol(data)) {
    counts[[i]] <- table(factor(data[,i], levels = 1:5))
  }

  lapply(counts, function(count) {
    # number of response categories, k
    k <- length(count)
    # set up mapping lists
    old <- as.numeric(names(count))
    new <- 1:k-1
    # note that the count variable is only used as a temporary bookkeeping
mechanism, old and new form the actual mapping.

    # loop over response cats, right to left up to second element.
    for (i in k:2){
      # if category is too low, add it to the left.
      if (count[i] < threshold){
        # update count for next step
        count[i-1] <- count[i-1] + count[i]
        count[i] <- 0

        # update new mapping
        new[i:k] <- new[i:k] - 1
      }
    }

    # first element
    if (count[1] < threshold){
      # sum first elements until threshold is reached
```

```
      for (i in 1:k) {
        if (sum(count[1:i]) >= threshold) break
      }

      # set counts
      count[i] <- sum(count[1:i])
      count[-(i:k)] <- 0

      # set new
      new[1:i] <- new[i+1] - 1
    }

    # start at zero
    new = new - min(new, na.rm = TRUE)

    if(length(unique(new)) == 1) warning("Only one response category left
after recoding.")

    list(old = old, new = new)
  })
}
```

In pseudo code, the steps are the following;

1) Convert response data into category counts
2) Starting from the highest response category, if the count is smaller than the threshold, collapse the category into the next lower category.
3) Repeat until the second-lowest category is reached.
4) If the count in the lowest category is smaller than the threshold, collapse the category into the next higher categories, until the threshold count is reached.
5) Finally, set the new mapping to start at zero.

The result is a list of mappings, one for each item. The actual recode is performed in a second step, with the aid of the recode() function from R package car (Fox & Weisberg, 2011).

References

Fox, J. and Weisberg, S. (2011) *An R Companion to Applied Regression*, Second Edition, Sage.
