# Supplementary material for: The COPD-SIB: a newly developed disease-specific item bank to measure health-related quality of life in patients with chronic obstructive pulmonary disease
Source: Health Qual Life Outcomes. 2016 Jun 27;14:97. doi: 10.1186/s12955-016-0500-0 (PMC4924274; doi:10.1186/s12955-016-0500-0)
Supplement: Additional file 4: — Calibration results of the final version of the COPD-SIB item bank. (PDF 294 kb) [file 12955_2016_500_MOESM4_ESM.pdf]

Supplement 4 Calibration results of the final version of the COPD-SIB item bank

Table S4 GRM estimates for the shortened (final) version of the COPD-SIB

Item nr	Theme	$\hat{\alpha}$	$\widehat{\beta_1}$	$\widehat{\beta_2}$	$\widehat{\beta_3}$	$\widehat{\beta_4}$
**1**	5	1.1	-2.54	-1.03	0.29	
**2**	5	1.18	-3.02	-0.9	0.31	1.51
**3**	5	1.45	-2.03	-0.26	0.71	1.82
**4**	5	1.47	-2.22	-0.3	0.77	1.61
**5**	5	1.25	-2.07	-0.37	0.62	1.88
**9**	8	1.76	-1.03	0.84	1.39	2.34
**14**	3	1.71	-2.47	-0.81	0.17	1.35
**15**	1	1.23	-2.23	0.02	0.94	2.68
**17**	2	1.49	-1.04	0.76	1.5	2.34
**18**	3	1.34	-2.66	-1.53	-0.36	1.18
**20**	1	1.42	-2	-0.4	0.64	2.36
**21**	3	1.89	-1.55	-0.41	0.69	
**22**	1	1.1	-0.99	-0.02	1.87	
**23**	1	2.26	-1.06	0.51	1.18	1.89
**25**	1	1.48	-2.22	0.15	0.82	2.18
**27**	3	1.98	-2.14	-0.84	-0.12	1.11
**28**	1	1.63	-1.29	0.58	1.32	2.41
**29**	2	2.1	-2.1	-0.43	0.02	1.18
**30**	8	1.74	-2.3	-0.63	-0.05	1.21
**32**	8	1.95	-2.02	-0.73	-0.17	0.93
**33**	8	1.76	-2.05	-0.73	-0.12	1.16
**34**	8	1.78	-1.34	-0.61	0.91	
**35**	8	1.36	-1.68	-0.48	1.03	
**36**	8	1.72	-1.78	-0.02	0.62	1.48
**37**	8	1.56	-0.52	0.22	1.62	
**38**	8	1.53	-1.84	0.08	0.72	1.89
**39**	8	1.44	-2.6	-0.73	0.1	1.55
**40**	8	2.02	-1.12	0.97	1.43	
**41**	8	1.14	-3.28	-1.16	-0.16	1.91
**42**	8	2.05	-2.16	-0.85	-0.15	1.19
**43**	8	2.59	-1.8	-0.61	0.03	1.14
**44**	8	1.63	-1.39	-0.37	1.61	
**45**	8	2.25	-1.76	-0.28	0.3	1.28
**46**	8	2.29	-1.94	-0.54	0.16	1.51
**47**	7	2.45	-1.09	0.13	0.63	1.63

**48**	7	1.53	-0.8	0.93	1.55	
**49**	7	2.25	-0.83	0.37	0.82	1.78
**50**	1	2.28	-2.01	-0.68	0.46	1.18
**51**	3	1.76	-2.35	-1.32	-0.62	
**52**	1	1.41	-2.06	-0.91	0.01	
**55**	3	1.93	-2.02	-0.84	0.26	
**56**	1	1.12	-3	-1.04	0.95	
**57**	1	1.35	-1.04	0.77		
**60**	6	1.89	-1.58	-0.2	1.6	
**62**	7	2.35	-1.43	-0.62	0.1	0.65
**63**	7	2.56	-2.02	-1.22	-0.18	0.47

Theme: 1 = Coping with disease/symptoms, adaptability; 2 = Autonomy; 3 = Anxiety about the course/end-state of the disease, hopelessness; 4 = Positive psychological functioning; 5 = Situations triggering or enhancing breathing problems; 6 = Symptoms; 7 = Activity; 8 = Impact
